# Dos and don’ts of a successfully peer-reviewed publication: From A–Z

**DOI:** 10.1556/1886.2020.00023

**Published:** 2020-10-03

**Authors:** PAYAM BEHZADI, MÁRIÓ GAJDÁCS

**Affiliations:** 1 Department of Microbiology, College of Basic Sciences, Shahr-e-Qods Branch, Islamic Azad University, Tehran, Iran; 2 Department of Pharmacodynamics and Biopharmacy, Faculty of Pharmacy, University of Szeged, Szeged, Hungary; 3 Institute of Medical Microbiology, Faculty of Medicine, Semmelweis University, Budapest, Hungary

**Keywords:** peer-review, medical manuscripts, microbiology publications, immunology publications, research, abstracting and indexing

## Abstract

To have a successful publication in a peer-reviewed journal, a collection of factors and items is needed. Some of them directly and the others indirectly have important roles in scholarly publication. However, a well-designed scientific investigation together with a powerful academic English language may guarantee the publication of a manuscript. In other words, a standard and professional methodology which is expressed by an influent academic English language constitutes the soul of the manuscript’s body. Obviously, the accuracy and fluency of the English language of the manuscript is the author(s)’ responsibility and neither the reviewers’ nor the editor’s and the journal’s. As publication of a research paper is the complementary section of a scientific study, it is recognized as an academic criterion for academicians. Thus, this review focuses on the all of items which are necessary and vital for a successful scholarly publication.

## INTRODUCTION

As references show, the year of 1665 was the first historical reference of writing modern scientific papers in scholarly journals. Although no style or standard format requirements were defined for scientific papers at that time, classification of formatted scientific papers was present even in the late 17th century [1]. Between 1665 and the 1850s, the scientific letters (which were authored by a single scientist with a polite summary of the literature regarding a variety of scientific topics) and experimental reports were the normal forms of scientific papers; from the 1850s, the structure of scientific papers evolved via the addition of the methods section into scientific papers. Thus, scientific papers from 1850s to 1900 were modernized by a methodology section to allow for the reader to ascertain the validity of the results and to reproduce the published experiments. The process of standardization in scientific publishing gained momentum begun from the year 1900 and it was solidified in the 1980s, by designing IMRAD structured original articles. Indeed, the acronym of IMRAD is the abbreviation for Introduction, Methods, Results, And Discussion [1–3]. Although many scientific fields (e.g., mathematics and theoretical physics among natural sciences or the humanities) may have their alternative guidelines on publishing results (book-like, newspaper-like scientific articles, Dunleavy’s structural alternatives), it is always the safest choice to use the IMRAD format in international journals [4]. Nevertheless, in case of special article types in medicine (e.g., PRISMA [Preferred Reporting Items for Systematic Reviews and Meta-Analyses] guidelines in case of systematic reviews and meta-analyses) additional special criteria must also be taken into consideration. As in many scientific fields, the quality and quantity of scientific publications by individual researchers is important for many reasons in medicine and also in the narrower fields of immunology, microbiology and infectious diseases. Young researchers pursuing a scientific career in these fields must comply with a set of rules by their doctorate school to be able to receive a Ph.D. (Doctor of Philosophy) degree, while in many clinical centers and university hospitals, clinical immunologists, infectious diseases specialists and clinical microbiologists must also publish scientific articles to pursue a promotion or leadership positions [5]. In accordance with our publishing experience and giving several workshops and lectures in this field and getting brilliant feedback by the participants, we decided to publish a paper discussing this topic in detail.

## THE PEER-REVIEW PROCESS: IMPORTANCE AND REASONS

As the quality and validity of scientific manuscripts are some of the most important qualities to consider; the number of peer-reviewed journals has significantly increased and their methods to rigorously evaluate scientific papers have also shown important progress in the last three centuries. Indeed, peer-reviewed journals evaluate the quality and validity of submitted scientific manuscripts by expert referees relating to the field. Usually, the scientific evaluation is achieved by determined reviewers (generally two or three referees) that are selected by the journal’s editor(s). The editor’s final decision is predominantly made in accordance with and based on the reviewers’ points of view; however, if discordant review reports are presented, it is the Editor’s responsibility to either sequester additional review reports or to make a decision by reflecting on the quality of the paper him/herself. The publication process of peer-reviewed journals is time-consuming as the validity and quality of scientific papers are evaluated by the professional specialists and experts who are usually also performing research and have educational responsibilities, therefore are busy [3, 6].

The evaluations performed by the reviewers may lead to the direct acceptance (publication in the present form), acceptance with minor revision, reconsider after major revision, weak rejection (resubmission after principal revisions) and direct rejection [3, 7]. Hence, the peer-review process – whether single-blind or double-blind – may act as a scientific filter; however there are some criticisms regarding the peer-review processes [8]. By the time and progression in publishing technology in the recent 200 years, peer-reviewed journals have had a flourishing progress [9]. On the other hand, after the 1990s, the introduction of online submission systems and the creation of electronic environments accelerated the speed of communication between editors, reviewers and authors even more, without the need of relying on paper-based communication and national/international postal systems. All these landmarks support the increase of submitted manuscripts and the reduction of the time needed for final decisions and revisions [10]. Hence, the publication period of manuscripts has decreased significantly during the last years, which is good news for the authors. However, we still hear from the inexperienced authors and novices that the process of a scientific publication is very hard. Why? This may be due to the fact that the majority of first-paper scientists think that the processes of writing a paper and performing scientific research are distinct professions, however, this is not the case. Scientific writing is a package of skills which an author should be trained for. In other words, writing a scientific paper is recognized as a blend of art and knowledge [3, 11].

In addition, authors must also have the ability to screen existing literature to find the appropriate place for their article, or to find the “gaps” of knowledge, which they may fill [12]. Therefore, the science behind scientific writing should be taught to beginners throughout training programs and workshops by experienced mentors, or as a part of graduate and postgraduate education. While in Western countries, this is usually already incorporated into the general (undergraduate) curricula, students in other countries first get acquainted with these competencies during PhD training [13, 14]. The publication of a *bona fide* scientific paper needs practice, experience, serenity, patience and an ability to accept failures and feedbacks, which are all integral parts of the peer-review process. As the mainstream scholarly peer-reviewed journals are predominantly in English, the language can be a serious problem for those authors that have English as their second language. Of course, the authors speaking English as their second language have lesser confidence in scientific writing than native English speakers [6, 15, 16]. The linguistic fluency and clarity is necessary in scholarly journals, because only an error-free text in academic English could be easily understood by the global readership [17, 18].

## PEER-REVIEWED JOURNALS AND THE RELATED METRICS

Scientific peer-reviewed journals are suitable and important means of spreading the results and outcomes obtained from scientific investigations [15, 19]. In addition to peer-review process, indexing and abstracting by scientific databases like EMBASE [1, 3], Clarivate Analytics (https://www.clarivate.com/) (Web of Science (WoS) [20]), MEDLINE [3, 7], Scopus [1, 3] and Science Citation Index [15] are important destinations for academic staff, graduate students and researchers to get their scientific works published with [7, 21]. Many universities only reward papers published in journals indexed in the abovementioned databases. Moreover, it is recommended to choose indexed journals with impact factor (IF) (or journal scientific impact), which indicates the level of citations of published articles by the journal in the last 2 years [8, 13]. However, the IF is not an all-round or exceptional indicator, but it is still used as a metric tool for increasing the quality of publications [7]. The IF for each journal is calculated as below [13]:

IF = the number of cited articles (all types) published by the journal in the last 2 years at present/the number of published articles (all types) by the journal in the last two years.

For example, the calculation of IF for a journal in 2018 gets back to the years of 2017 and 2016.

The IF of a journal from Journal Citation Reports (JCR) may be found on the website of the database (https://clarivate.com/products/journal-citation-reports/). It is recommended to check the JCR for your interested journal if there is any suspicious regarding the journal’s IF [22].

Rather than the IF, other indicators including CiteScore (https://www.elsevier.com/editors-update/story/journal-metrics/citescore-a-new-metric-to-help-you-choose-the-right-journal), the Eigenfactor Score (ES) (http://www.eigenfactor.org/projects/journalRank/journalsearch.php), Article Influence Score (AIS), SCImago Journal Rank (SJR) (https://www.scimagojr.com/) [21, 23], Immediacy index [21], IF^2^-index (the Impact Factor squared) [24] and Hirsch index (H-index) [21] are also important items to measure the validity and influence of the journals. The CiteScore metrics belongs to Scopus journal metrics (https://www.scopus.com/sources) and is calculated just like the IF but for three previous years ago. For instance, the CiteScore of a journal in 2018 goes back to the years of 2015, 2016 and 2017 (https://www.elsevier.com/editors-update/story/journal-metrics/citescore-a-new-metric-to-help-you-choose-the-right-journal).

The ES is supported by Institute for Scientific Information (ISI) while the SJR is covered by Scopus [23]. Another important indicator of the scientific quality of a journal is their quartile (Q) ranking, which corresponds to a categorization of each journal in each of its subject categories (which was previously set by the journals, based on their scope), according to which quartile of the IF distribution the journal occupies for that particular subject category. In many cases, academic institutions in Microbiology and Immunology favor scientists to publish in Q1 journals in the subject areas of “Immunology and Microbiology”, or in a more specific subject category, based on the scope of the scientists’/physicians’ activities. In these fields, the following narrower subject categories are available: i. applied microbiology and biotechnology, ii. immunology, iii. immunology and allergy, iv. immunology and microbiology (miscellaneous), v. microbiology, vi. microbiology (medical), vii. infectious diseases, viii. parasitology and ix. virology [23]. For scientists working in the subject areas of immunology and microbiology, it is desirable to check the quartile ranking of journals to see, whether they are indexed in some of the abovementioned subject categories. Nevertheless, it should be noted that many journals are indexed in multiple subject areas and categories, and they may hold significantly different places in quartile rankings (e.g., Q1 in “Public Health, Occupational and Environmental Health”, Q2 in Medicine, but Q3 in “Microbiology”), which is due to the different journal composition in each individual category and the corresponding impact factor values. Generally, IF values of microbiology and immunology journals (especially if the scope includes clinical aspects) are among the highest in the list of specialized journals [23–25]. The evaluation process of papers published in these “mixed” category journals by university bodies is usually on a case-by-case basis. However, none of these indicators can be considered as an independent gold standard quantitative metrics.

According to some estimation, there are up to 10,000 journal publishers worldwide that cover different numbers of journals. Among a wide range of journal publishers, some largest journal publishers, Springer (with 3,591 journals) [8] (https://rd.springer.com/search?facet-content-type=%22Journal%22), Wiley (with 2,565 journals) (https://onlinelibrary.wiley.com/action/showPublications?PubType=journal&startPage=0&pageSize=20), Elsevier (with 2,536 journals) (https://www.elsevier.com/catalog?producttype=journals&cat0=&q=&search=1&imprintname=&categoryrestriction=&sort=datedesc), Taylor and Francis (with over 2,200 journals) (https://www.scelc.org/offers/taylor-and-francis-journal-packages), Oxford University Press (OUP) (with 453 journals) (https://academic.oup.com/journals/pages/journals_a_to_z) are known as the largest publishers of important and well-known academic journals. Prior to starting a journal’s draft, an author should raise the following pivotal questions from yourself: “What is the importance of my work?” and “What is novelty of my work?”. If you have considerable answers to these questions, design your manuscript’s blueprint and be ready to write your paper to publish it [3, 6, 15];otherwise forget it and begin another proposal with a strong roadmap.

## DOS AND DON’TS OF A SUCCESSFUL PUBLICATION

In order to successfully publish a scholarly paper, there are some items which should be considered. The authors should be aware of the importance of peer-reviewed publications; indeed, publication of scientific papers in scholarly journals is known as an academic credit and career promotion [1, 16, 24]. A strong scientific paper is like a healthy and strong human. A suitable and favorite mold and format of the paper is similar to the body and the well-designed study of the paper resembles the soul of a healthy and strong human. While there are some stylistic and formatting concerns that are universal (e.g., adhering to conventions regarding the writing of bacterial names or geographical locations [26]), many instructions vary from journal to journal therefore, it is the authors’ responsibility to adhere to these guidelines; non-adherence will usually lead to immediate rejection. Furthermore, the improper reporting style (including the use of Jargons and unfavorable terminology) and poor academic English are also considered as common reasons for direct rejection. English language polishing is a must to publish a scientific paper in a peer-reviewed scholarly journal [1, 2, 6, 13, 15]. Obviously, the accuracy and fluency of the English language of the manuscript is the author(s)’ responsibility and reviewers’ nor the editor’s and the journal’s. Hence, it is recommended that the non-native English speakers to have a through proof-reading by a native English speaker.

Using short and simple sentences are the best means for transferring a paper’s data and information to the readers. Repetition, unclear, weak and poorly understandable sentences are hidden means for direct rejection [7,22,27,28]. In addition to these aforementioned items, to have a successful scholarly publication there are other important considerations which should be taken into account as well. The authors should select their journal(s)ofinterest and check it if the journal has an IF and is indexed by some known international and important databases including MEDLINE [1, 3], WoS [20] and Scopus [1, 3]. For many years, the authors had to look for and select a target journal by themselves; and as we know, this process is time-consuming and may be overwhelming. Fortunately in recent years, large scholarly publishers including Elsevier (https://journalfinder.elsevier.com/), Springer (https://journalsuggester.springer.com/) and Wiley (https://journalfinder.wiley.com/search?type=match) have prepared journal finder systems which can be used for choosing proper journal(s). These systems are very helpful because Scopus, Springer and Wiley indexed Journals involve a huge number of peer-reviewed journals including 38,380 journals (active and inactive) [29], 3,596 journals (https://rd.springer.com/search?facet-content-type=%22Journal%22) and 1,493 journals (https://libopac.josai.ac.jp/search/journal/WileyOnlineLibrary.pdf), respectively. Besides, these journal finder systems –depending on the related publishers-represent important information about the introduced journals including IF, ISI ranking, CiteScore, publication type, journal’s ISSN, average time for the journal’s first decision, average time for the publication, acceptance rate, relevance and direct access to the journal’s website. This facility is a great opportunity for the authors to decide which journal is suitable to submit their manuscripts. After finding suitable journal(s), the authors should read the “Aims and Scope” of the journal to avoid immediate rejection. We believe that the authors should be aware from the scientific level of their manuscript and the selected journal. We, as professional reviewers have observed several manuscripts with well-designed methodology and fluent academic English which were submitted to the wrong journal. This status will lead to direct rejection. The authors should take into account that a prestigious peer-reviewed journal employs expert referees.

When you receive the referees’ comments, read them word by word and answer them in polite and scientific manner. Then, consider the requested materials within the text one by one; and finally, highlight the addenda. A professional author never debates with the referee(s); because the losers are the authors of the manuscript. The reviewers try to improve the quality of your manuscript; therefore it is important to just consider the reviewers’ comments carefully and be thankful for their helpful recommendations [1, 28, 30]. Sometimes, the reviewer’s decision is direct rejection; however, don’t give up! Consider the related improvements and choose another journal [1, 28, 30].

## CHOOSING THE APPROPRIATE JOURNAL

As the publication process of a paper in a peer-reviewed journal is hard and time-consuming, novices try with those journals that publish their work in a short period of time (for a limited amount of money). Many of these types of journals are known as predatory journals. Jeffrey Beal, an associate professor at Denver University, Colorado, and the Scholarly Communications Librarian coined the term of “Predatory” journals. In 2008, when the number of these doubtful journals was considerably increasing, he actively investigated regarding these predatory journals (other names: dark journals, deceptive journals, illegitimate journals, journals operating in bad faith) [31, 32]. Predatory journals bombard authors from different regions and countries (their targets are mostly the authors from countries with low and middle incomes) by sending a huge number of e-mails (called as “fishing” e-mails) with low publication fees. In other words, the predatory journals are discounted open-access journals which publish low quality papers during a short time [31-35]. In accordance with previous studies [9, 32] the average period of publication in predatory journals is 2.7 (from submission to publication) while this period for peer-reviewed journals may be >12 months. This comparison reveals the lack or the weakness of peer-review process in predatory journals [9, 32].

There are different resources for detecting predatory journals including Beall’s list (down on January 15, 2017), Cabell’s International (http://www2.cabells.com/about-blacklist), Think. Check. Submit. (https://thinkchecksubmit.org/) and Queen’s University Library (https://guides.library.queensu.ca/deceptive-pubs-conf/checklist) websites [32, 33, 36, 37]. In accordance with Predatory Publishers Checklist represented by Queen’s University Library, there are several items for recognition of predatory journals. Among them, not being a member of the following recognized scholarly organization including DOAJ (Directory of Open Access Journals) (https://doaj.org/), ICMJE (International Committee of Medical Journal Editors) (http://www.icmje.org/), COPE (Committee on Publication Ethics) (https://publicationethics.org/), INASP (for journals published in Bangladesh, Nepal, Sri Lanka, Central America and Mongolia) (https://www.inasp.info/project/journals-online-project), OASPA (Open Access Scholarly Publishers Association) (https://oaspa.org/), AJOL (African Journals Online) (https://www.ajol.info/) and WAME (World Association of Medical Editors) (http://www.wame.org/) can be used as a suitable criterion for detecting predatory journals (https://guides.library.queensu.ca/deceptive-pubs-conf/checklist) [32, 34, 35, 38].

Avoiding self-plagiarism, “salami publication” (i.e. wasteful publication), pure plagiarism (copy-and-paste), paraphrasing, falsification, fabrication, duplication, sending a manuscript to two peer-reviewed journals at the same time and so on are the red-lines for the authors for not being blacklisted by the academic journals [1–3, 28, 39–42]. The authors are recommended to go to COPE website (https://publicationethics.org/) for ethical issues in details. The COPE’s core practices include allegations of misconduct, authorship and contributorship, complaints and appeals, conflicts of interest, data and reproducibility, ethical oversight, intellectual property, journal management, peer-review processes, and post-publication discussions (https://publicationethics.org/). The ICMJE guidelines include recommendations (relating to conduct, edition, publication and reporting of scientific investigations and studies), conflicts of interest, journals, news and editorials regarding medical journals (http://www.icmje.org/).

## CONCLUSIONS

Due to the technical progression of journals’ submission systems, each journal receives a huge number of manuscripts around the world. Due to this facility, the peer-reviewed and quality journals try to select the best articles for publication. Thus, to have a successful publication in a peer-reviewed and indexed scholarly journal with an IF, a well-designed study, strong methodology and skillful author is needed, with high ability to write scientific paper in effective and fluent academic English language. These types of authors are able to satisfy Editors-in-Chief and reviewers of high quality journals, resulting in a subsequent publication of a paper. To be a skillful author needs practice, practice, practice and patience!
